# Healthcare needs, experiences and treatment burden in primary care patients with multimorbidity: An evaluation of process of care from patients' perspectives

**DOI:** 10.1111/hex.13363

**Published:** 2021-09-28

**Authors:** Xiu‐Jing Hu, Harry H. X. Wang, Yu‐Ting Li, Xiao‐Ya Wu, Yi Wang, Jia‐Heng Chen, Jia‐Ji Wang, Samuel Y. S. Wong, Stewart W. Mercer

**Affiliations:** ^1^ School of Public Health Sun Yat‐Sen University Guangzhou China; ^2^ JC School of Public Health and Primary Care, Faculty of Medicine The Chinese University of Hong Kong Shatin Hong Kong SAR; ^3^ State Key Laboratory of Ophthalmology, Zhongshan Ophthalmic Center Sun Yat‐Sen University Guangzhou China; ^4^ Guangdong‐provincial Primary Healthcare Association Guangdong China; ^5^ School of Public Health Guangzhou Medical University Guangzhou China; ^6^ Centre for Population Health Sciences, Usher Institute University of Edinburgh Scotland UK

**Keywords:** health services evaluation, multimorbidity, patients' experiences, Primary Care Assessment Tool (PCAT), process of care, Treatment Burden Questionnaire (TBQ)

## Abstract

**Background:**

Patients with multimorbidity often experience treatment burden as a result of fragmented, specialist‐driven healthcare. The ‘family doctor team' is an emerging service model in China to address the increasing need for high‐quality routine primary care.

**Objective:**

This study aimed to explore the extent to which treatment burden was associated with healthcare needs and patients' experiences.

**Methods:**

Multisite surveys were conducted in primary care facilities in Guangdong province, southern China. Interviewer‐administered questionnaires were used to collect data from patients (*N* = 2160) who had ≥2 clinically diagnosed long‐term conditions (multimorbidity) and had ≥1 clinical encounter in the past 12 months since enrolment registration with the family doctor team. Patients' experiences and treatment burden were measured using a previously validated Chinese version of the Primary Care Assessment Tool (PCAT) and the Treatment Burden Questionnaire, respectively.

**Results:**

The mean age of the patients was 61.4 years, and slightly over half were females. Patients who had a family doctor team as the primary source of care reported significantly higher PCAT scores (mean difference 7.2 points, *p* < .001) and lower treatment burden scores (mean difference −6.4 points, *p* < .001) when compared to those who often bypassed primary care. Greater healthcare needs were significantly correlated with increased treatment burden (*β*‐coefficient 1.965, *p* < .001), whilst better patients' experiences were associated with lower treatment burden (*β*‐coefficient −0.252, *p* < .001) after adjusting for confounders.

**Conclusion:**

The inverse association between patients' experiences and treatment burden supports the importance of primary care in managing patients with multimorbidity.

**Patient Contribution:**

Primary care service users were involved in the instrument development and data collection.

## 1 INTRODUCTION

Multimorbidity—the presence of two or more chronic conditions within an individual—has become increasingly common over recent decades.^1^, ^2^, ^3^, ^4^ It presents complex challenges to patients, such as functional decline, mental health difficulties, polypharmacy, reduced quality of life, increased hospital admission and risk of severe COVID‐19.^5^, ^6^, ^7^, ^8^, ^9^, ^10^ Existing evidence supports the role of high‐quality primary care in improving population health outcomes in a cost‐effective manner, and primary care is of particular importance in addressing multiple healthcare needs.^1^, ^11^

China, like many countries that are facing health and social care challenges from an ageing population, is reshaping its healthcare system with a primary care‐oriented approach to pursue equitable population health and reduce the burden of chronic conditions.^12^, ^13^ Primary care facilities have been established for delivering safe, effective, convenient and affordable healthcare by general practice (GP) physicians outside of hospitals. However, healthcare gatekeeping is largely absent and thus people can bypass primary care and go straight to hospitals for specialist care as they wish. The concept of a ‘family doctor team' has been gradually translated into practice since June 2016 as an emerging healthcare model built on the national basic public health (BPH) service package.^14^, ^15^ A typical team is comprised of one GP clinician and several healthcare personnel including nurses, public health doctors and, if available and suitable, pharmacists and social workers. This supports a broader range of systematic preventive care approaches, including health assessment, health promoting interventions, health advice and, when necessary, home visits to support self‐management. The primary care multidisciplinary team is expected to be responsible for the health of enrolled people and their family members.^15^, ^16^

The management of a population with multimorbidity requires routine primary care that is respectful of, and responsive to, their increasing need for family‐centred continuity of care as opposed to hospital‐based fragmented care. In a fragmented healthcare system, it is less likely that multiple, episodic healthcare providers will take into account the entirety of a patient's healthcare conundrum including inappropriate polypharmacy, demanding self‐management regimens and competing priorities and more vulnerability to safety issues due to multimorbidity.^3^ This would inevitably lead to unaddressed issues associated with greater lapses in quality and safety, higher healthcare expenditure and more avoidable hospital admission.^9^, ^17^ Management of multimorbidity is complex and necessitates coping strategies built upon continuous care with consultations, examinations, medications and lifestyle changes, placing significant burden on patients in terms of excessive time, efforts and attention.^4^, ^18^, ^19^, ^20^ The understanding of processes of care that take into account patients' healthcare needs and minimize treatment burden is an essential step to inform service delivery for multimorbidity.^21^ These relationships between need, patients' experiences, treatment burden and use of primary care have not been described before, and are highly relevant in the context of the growing challenge of multimorbidity globally.

This study aimed to provide an insight into healthcare needs, patients' experiences and treatment burden from the perspective of process of care. Our key research question is whether there is a significant association between primary care experiences and treatment burden in the context of patients' increasing healthcare needs due to multimorbidity. In the absence of a secondary healthcare gatekeeping function in primary care, we hypothesize that patients who do not consider the family doctor team as their preferred usual source of care will have poorer primary care experiences and greater treatment burden.

## 2 METHODS

### 2.1 Study design

Multisite cross‐sectional survey data were collected from primary care service users, with a diversity of geographic locations, in 9 out of a total of 21 cities in Guangdong province, southern China. In the first stage, three cities were selected in each of the western, central and eastern areas of Guangdong, respectively. In the second stage, two sites per city were randomly selected from primary care facilities that were organizational members of the Guangdong Primary Healthcare Association to facilitate the fieldwork coordination.

### 2.2 Setting and data source

The study was conducted on‐site at 18 primary care facilities where free‐of‐charge, annual check‐up, as part of the national BPH service package, was offered to people aged ≥35 years who had hypertension or diabetes.^14^ Routine primary care patients who fulfilled the eligibility criteria were invited on the day of their check‐up visits at community health centres (CHCs). Our previous work showed that a minimum of 2500 community residents had enrolment registration with the CHC family doctor team,^12^ and that more than 10% of the general population had ≥2 chronic conditions (multimorbidity).^2^ We assumed a check‐up attendance rate of at least 60% and a survey response rate of no less than 80%. This yielded a sample size of 2160—that is, 120 participants recruited in each CHC. Interviewer‐administered questionnaires including items derived from our previous research^2^, ^22^ were used to collect data on demographics, socioeconomic status, health characteristics, healthcare needs, service utilization and the process of care from study participants.

### 2.3 Participants

The inclusion criteria of target participants were as follows: (1) patients who had ≥2 clinically diagnosed long‐term conditions including hypertension or type 2 diabetes and (2) had at least one clinical encounter in the past 12 months since enrolment registration with the family doctor team. We excluded those who were passers‐by (i.e., patients who were not enrolled or only recently enrolled with the family doctor team) to ensure that all study participants had valid exposure to the primary care provider before study participation, and could hence minimize the likelihood of capturing ‘hearsay' information that was not actually experienced by the patients. Patients who were unable to communicate or who were not on regular medications were excluded. Eligible participants were referred to trained interviewers by healthcare staff, following a modified systematic random sampling that was previously used.^22^

### 2.4 Measurements of patients' experiences

Patients' experiences were captured by a previously validated, culturally adapted, Mandarin Chinese version of the Primary Care Assessment Tool (PCAT)‐Adult Edition used in our previous research.^13^, ^22^ The instrument measures nine primary care attributes, that is, the first‐contact accessibility and utilization (first‐contact domain), continuity of care (longitudinal domain), coordination of services and information system (coordination domain), comprehensiveness of service availability and provision (comprehensiveness domain) and community orientation and family centredness (derivative domain).^23^ First‐contact care accessibility refers to whether patients are able to receive primary care whenever needed within a reasonable time in nonemergency situations, whereas first‐contact care utilization measures the extent to which a gatekeeper function is performed by the primary care provider. Coordination of care services assesses the linkage of healthcare visits across different levels in the health system, whereas the information system coordination measures the availability of health records for patients. All individual items were scored on a 4‐point Likert‐type scale, with higher scores indicating more positive experiences.^22^, ^23^ The total PCAT scores were calculated by summing up values from each of the nine scales. An adapted algorithm from the PCAT guideline was used to identify respondents' usual source of care, including both frequent and less frequent primary care service users.^22^

### 2.5 Measurements of treatment burden

Treatment burden was defined as the challenges that patients face in coping with everything they have to do to take care of their health, and its impact on functioning and well‐being.^20^, ^24^, ^25^, ^26^ It involves a variety of treatment workloads pertaining to medication management, self‐monitoring, laboratory tests, doctor visits, need for organization, administrative tasks, lifestyle changes and social impact.^27^, ^28^ The Treatment Burden Questionnaire (TBQ) is one of several existing measures, but was specifically developed to assess treatment burden among patients with multiple chronic conditions.^28^ It is composed of 15 items using a 10‐point rating scale, ranging from 0 (not a problem) to 10 (big problem). The sum of all item scores was calculated, and higher scores indicated greater treatment burden.^28^ A total TBQ score of 59 is a recommended cut‐off for defining high burden.^26^ A Mandarin Chinese version of the TBQ instrument was developed by our team (TBQ_AU1.0_cmn‐CN_RC, commissioned by the Mapi Research Trust) following a standard forward‐and‐backward translation methodology. The linguistic congruence and cultural relevancy were assessed on an item‐by‐item basis by a review panel consisting of two frontline GP physicians with over 10 years of working experiences and ten primary care adult patients with multimorbidity. Cultural differences in language usage were explored, with minor adaptations made to ensure the cultural relevance and contextual appropriateness for the implementation of TBQ in mainland China, while maximizing the equivalence of translation at the same time. Our further evaluation of psychometric properties suggested a good reliability and validity of TBQ for measuring treatment burden in the Chinese patients. The component matrix yielded from factor analysis explained 71.3% of the total variance. The overall Cronbach's *α* was .884, suggesting satisfactory internal consistency reliability. The test–retest intraclass correlation coefficients of individual item scores ranged from .725 to .846, suggesting that the results of assessment were stable through repetition.

### 2.6 Content validity and interviewer training

The content validity of the entire questionnaire, including both PCAT and TBQ instrument items, was assessed by a panel consisting of two GP professionals (J.‐J. W. and X.‐Y. W.) and two public health specialists (H. H. X. W. and Y.‐T. L.). Each item was rated with regard to the relevancy and clarity by a content validity index using a 4‐point Likert‐type scale. All items were rated as quite (3‐point) or highly (4‐point) relevant and clear by all panel experts. Survey interviewers included on‐site healthcare staff and medical university students. Training sessions were held by the two lead investigators. The survey was pilot‐tested by paired interviewers among 20 primary care service users to improve the interrater reliability.

### 2.7 Statistical analysis

Data entry was independently performed by two trained medical students using EpiData 3.1 with double verification. Sample mean with standard error (SE) or 95% confidence interval (CI), where appropriate, was applied in descriptive analysis. *χ* ^2^ tests or Student's *t* tests, where appropriate, were used to compare the differences with regard to categorical and continuous variables between groups. A general linear model analysis was conducted to examine patient‐level factors associated with treatment burden after controlling for confounders. The absence of multicollinearity and plausible interactions among variables were tested to ensure the robustness of the linear regression model. We also performed a series of sensitivity analyses to further explore the relationship between primary care experiences and each treatment burden measure, while controlling for other confounding factors in the multiple linear regression analysis. A *p*‐value <.05 was considered statistically significant. All statistical analyses were performed in IBM SPSS Statistics 25, and the Complex Samples module was used to account for the multistage sample design.

### 2.8 Ethics consideration

All study participants provided written consent. Data anonymization was performed by removing all patient identifiers from the data set before data analysis. Ethics approval was granted from the School of Public Health Biomedical Research Ethics Review Committee at Sun Yat‐Sen University (SYSU‐SPH2016027) in accordance with the Declaration of Helsinki 2013.

## 3 RESULTS

### 3.1 Characteristics of the study participants

A total of 2160 out of 2471 eligible primary care patients with multimorbidity were included (overall response rate 87.4%). The mean age of the participants was 61.4 years (95% CI: 60.7–62.2 years), and slightly over half were females. Less than half had completed secondary school education or above. Nearly one in five patients had multimorbidity for over 10 years. Approximately 40% of people had a monthly household income per capita below ¥2000. When compared to China's median disposable personal income (¥2028 per month) in 2018,^29^ the study participants were relatively wealthier than the general population (Table 1).

**Table 1:** Characteristics of the survey participants with multimorbidity

Variables	*N*	% (95% CI)
Sociodemographic characteristics		
Age groups (years)		
35–49	501	23.2% (21.2–25.4)
50–64	723	33.5% (31.5–35.5)
65–79	719	33.3% (30.9–35.7)
80 and above	217	10.0% (8.8–11.4)
Gender		
Male	951	44.0% (42.1–46.0)
Female	1209	56.0% (54.0–57.9)
Education level		
Primary school or blow	1194	55.3% (51.6–58.9)
Secondary school and above	966	44.7% (41.1–48.4)
Presence of social medical insurance		
No/out‐of‐pocket payment	200	9.3% (7.8–11.0)
Yes/insured	1960	90.7% (89.0–92.2)
Monthly household income per head		
Less than ¥2000	868	40.2% (38.3–42.1)
¥2000–4999	1104	51.1% (49.1–53.1)
¥5000 and above	188	8.7% (7.5–10.1)
Lifestyle behaviours and health conditions		
Cigarette smoking		
Current smoking	407	18.8% (17.0–20.8)
Noncurrent smoking	1753	81.2% (79.2–83.0)
Alcohol consumption		
Regular drinking	344	15.9% (14.5–17.5)
Nonregular drinking	1816	84.1% (82.5–85.5)
Duration of chronic conditions (years)		
<5	934	43.3% (41.6–44.9)
5–10	817	37.8% (36.5–39.2)
>10	409	18.9% (17.4–20.6)

*Note*: Monthly income levels were categorized according to the National Bureau of Statistics, PRC. Available at: http://www.gov.cn/xinwen/2019-01/25/content_5361066.htm

Abbreviation: CI, confidence interval.

### 3.2 Profile on service utilization and healthcare needs

**Figure 1:**
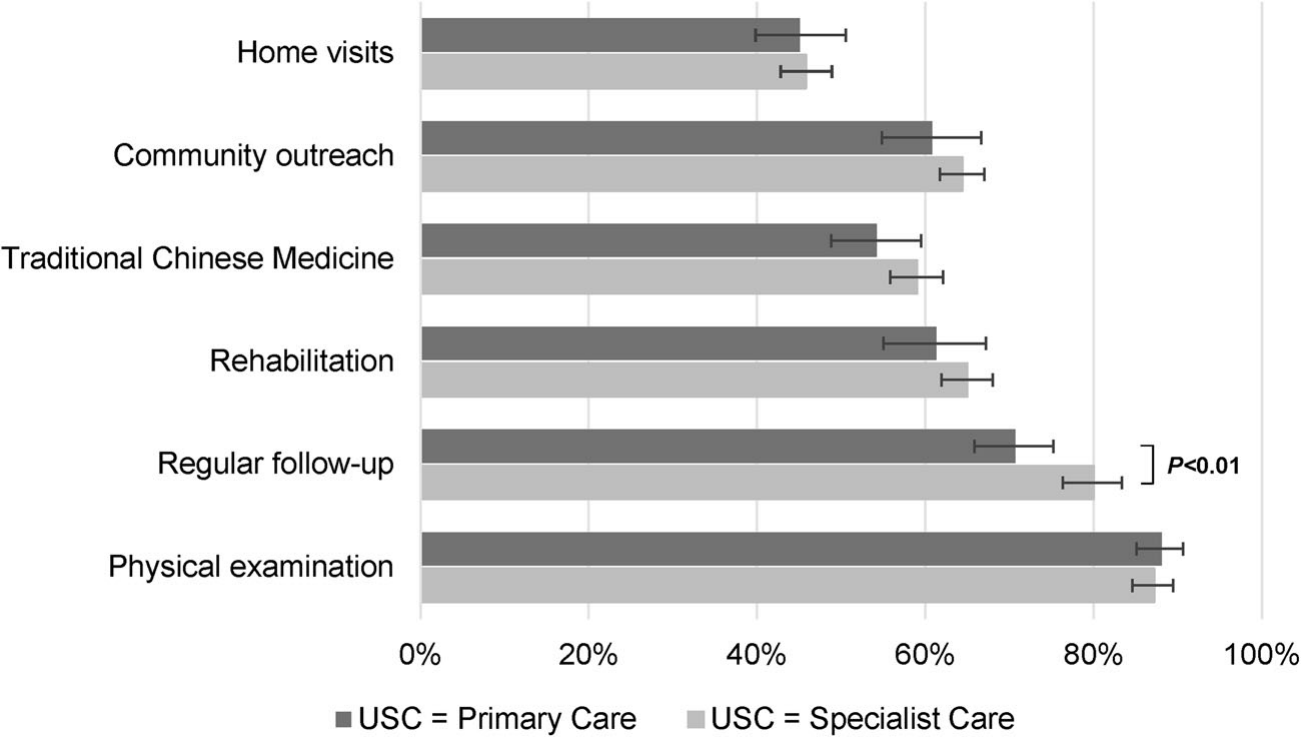
Self‐perceived healthcare needs in patients with multimorbidity. USC, usual source of care

More than two thirds (70.1%) of the participants considered the CHC family doctor team as their usual source of primary care. The traditional face‐to‐face visit was more common than distance communications for consultations. The family doctor team played a moderate positive role in service delivery perceived by patients, ranging from 53.7% to 68.1%. On average, the duration of CHC enrolment registration was 11.8 months, and each patient received 3.6 follow‐up appointments annually (Table 2). Participants who used specialist care rather than primary care as their usual source of healthcare reported a greater need for follow‐up care (mean difference 10.0%; *p* = .001) when compared to their counterparts (Figure 1).

**Table 2:** Profile on the process of primary care among the study participants

Variables	
Categorical	*N* (%)
Usual source of care	
CHC (primary care)	1514 (70.1%)
Hospital (outpatient specialist care)	646 (29.9%)
Usual channels to interactive consultations^a^	
Personal visits to CHCs	1386 (64.2%)
Distance communications	1042 (48.2%)
Patients' perceived roles of family doctor team in service delivery^a^	
Expanded coverage of prevention and treatment	1350 (62.5%)
Reduced expenditure on medical care	1160 (53.7%)
Improved access to healthcare	1470 (68.1%)
Continuous	Mean (SE)
Duration of CHC visits (months)	11.8 (1.5)
Frequency of follow‐up, times per year	3.6 (0.3)
Patients' experiences (PCAT; range of values)	
First contact: utilization (3–12)	9.91 (0.08)
First contact: accessibility (4–16)	9.97 (0.26)
Continuity of care (4–16)	11.57 (0.15)
Coordination of services (4–16)	11.51 (0.11)
Coordination: information system (3–12)	9.76 (0.18)
Comprehensiveness: services available (4–16)	12.48 (0.14)
Comprehensiveness: services provided (5–20)	16.62 (0.09)
Family centredness (3–12)	9.48 (0.08)
Community orientation (3–12)	8.76 (0.14)
PCAT total score (33–132)	100.70 (0.92)
Treatment burden (TBQ; range of values)	
TBQ total score (0–150)	43.90 (0.86)

Abbreviations: CHC, community health centre; PCAT, Primary Care Assessment Tool; SE, standard error; TBQ, Treatment Burden Questionnaire.

^a^ Sum‐up exceeds 100% as participants may choose more than one option.

### 3.3 Treatment burden and patients' experiences with primary care

**Figure 2:**
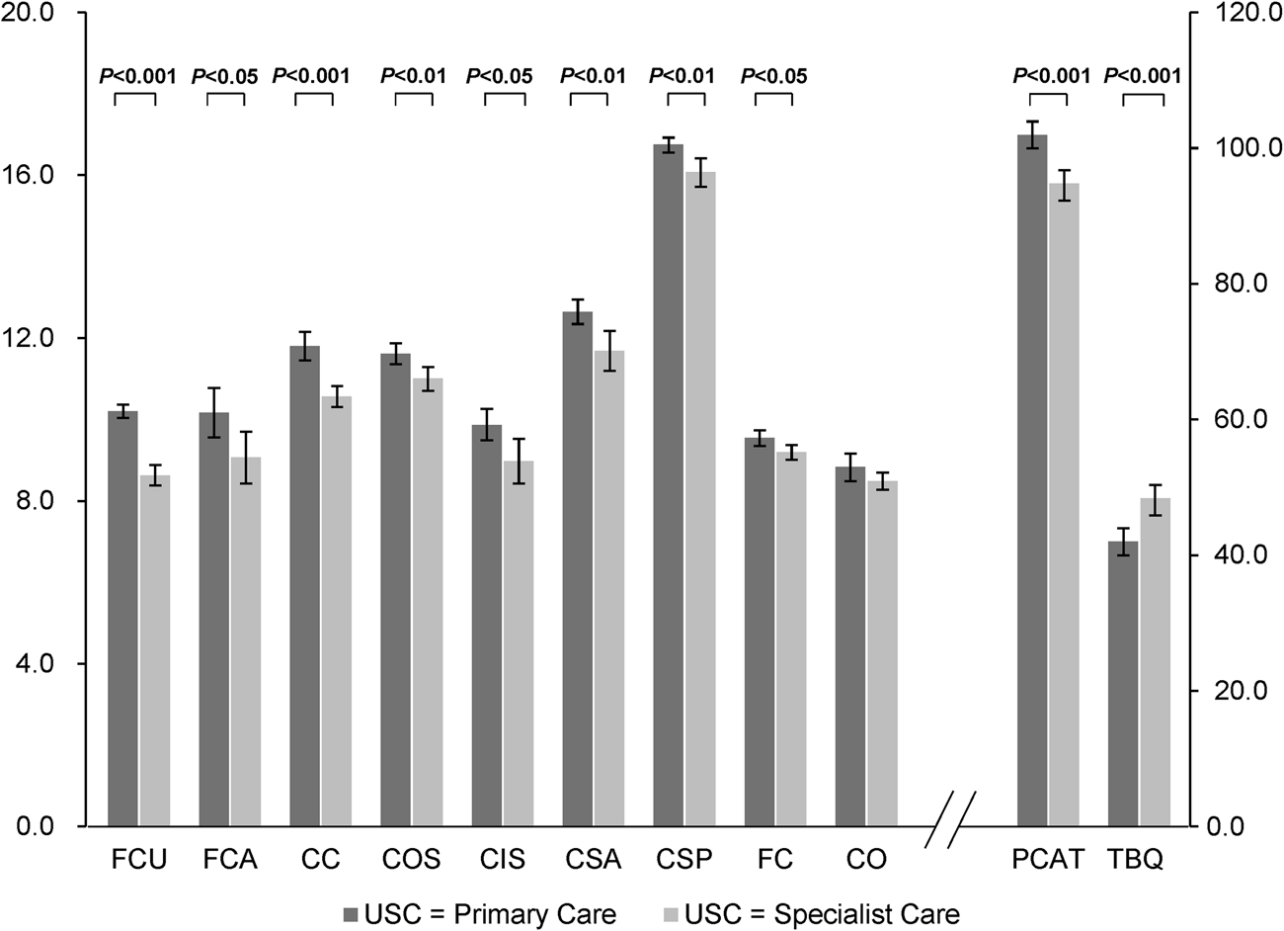
Comparison of primary care assessment and treatment burden. Note: Error bars indicate 95% confidence intervals of score means. CC, continuity of care; CIS, coordination (information system); CO, community orientation; COS, coordination of services; CSA, comprehensiveness (services available); CSP, comprehensiveness (services provided); FC, family centredness; FCA, first contact (accessibility); FCU, first contact (utilization); PCAT, Primary Care Assessment Tool (total score); TBQ, Treatment Burden Questionnaire (total score); USC, usual source of care

Participants reported an average global treatment burden score of 43.9 (SE: 0.9), which was slightly higher than the first quantile of the score range (0–150), whilst a total primary care assessment score of 100.7 (SE: 0.9) was reported on average, falling within the third quantile of the score range (0–132). This implied moderate‐to‐light treatment burden and medium‐to‐optimal primary care experiences overall (Table 2). Significant differences existed across most of the individual primary care scales between groups. In particular, patients who considered the family doctor team as the primary source of care had significantly better patients' experiences (mean difference 7.2 points, 95% CI: 4.6–9.8, *p* < .001) and lower treatment burden (mean difference −6.4 points, 95% CI: −9.6 to −3.1, *p* < .001) when compared to their counterparts who were in favour of using specialist care over primary care (Figure 2).

### 3.4 Factors associated with treatment burden

**Figure 3:**
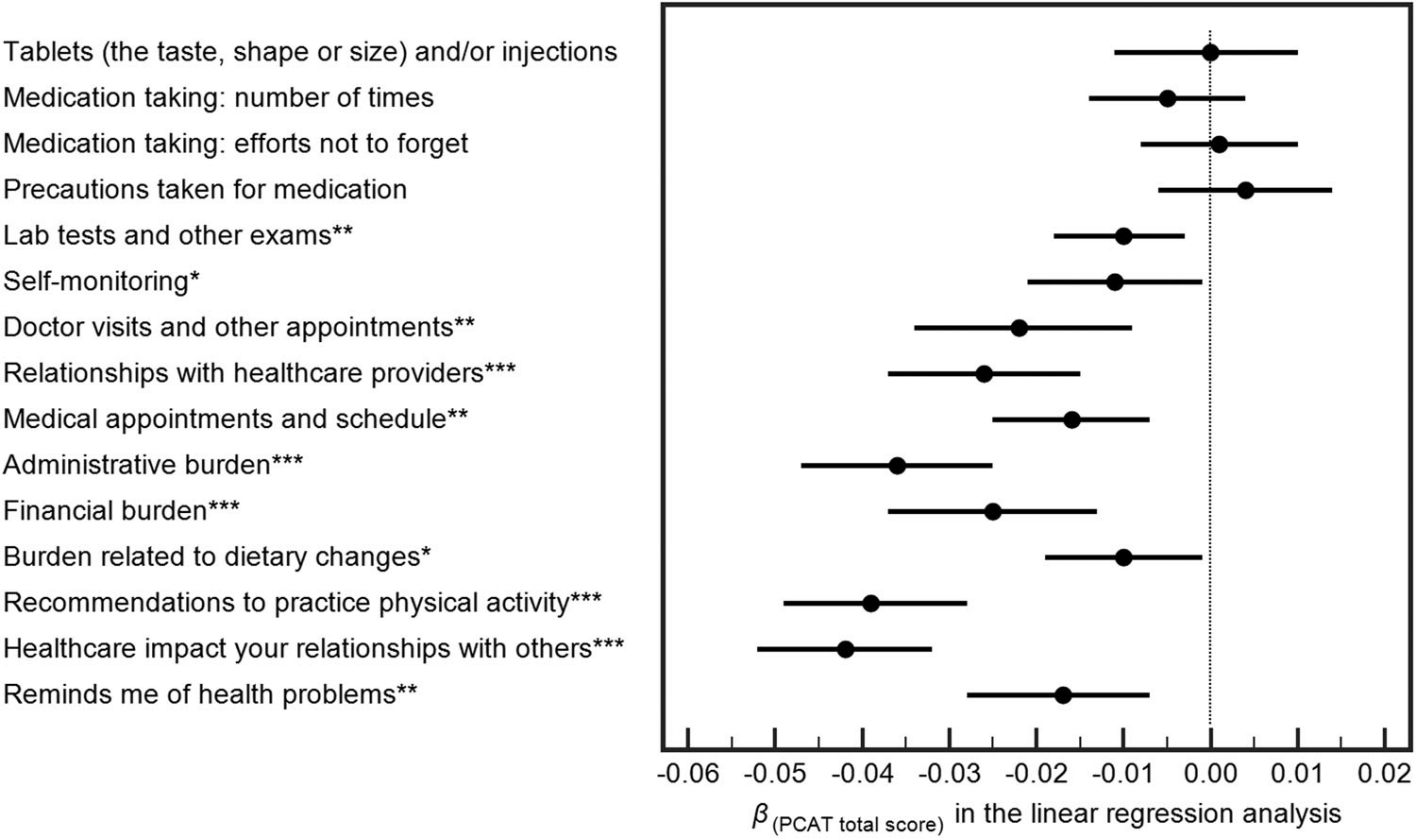
Association between the overall primary care assessment (independent variable) and each treatment burden measure (dependent variable) in the multiple linear regression analysis. Note: PCAT, Primary Care Assessment Tool; TBQ, Treatment Burden Questionnaire. Error bars indicate 95% confidence intervals of the *β*‐coefficients for the PCAT total score (i.e., independent variable; *X*) in each regression model with regard to each individual item score in the TBQ (i.e., dependent variable; *Y*), respectively, while controlling for other confounding factors that were statistically significant in the general linear model analysis shown in Table 3. *p < .05; **p < .01; ***p < .001

In the unadjusted model, variables pertaining to healthcare needs, service utilization and patients' experiences were all significantly associated with treatment burden. After controlling for all other variables in the regression model, the directions of significant associations remained unchanged, although the strengths were slightly attenuated. Longer duration of diseases (*β*‐coefficient 2.430, 95% CI: 0.808–4.051, *p* = .004) and increased counts of healthcare needs (*β*‐coefficient 1.965, 95% CI: 1.384–2.545, *p* < .001) were positively associated with greater treatment burden. Factors negatively associated with treatment burden included frequently delivered follow‐up (*p* = .001), regular use of primary care (*p* = .007) and higher primary care assessment scores (*p* < .001), indicating that better patients' experiences were associated with lower treatment burden overall (Table 3). Moreover, in the sensitivity analysis with each individual TBQ item score as the dependent variable in the regression analysis, the negative associations between primary care experiences and treatment burden were consistently observed, except for medication‐related item scores, albeit that the lower boundary of the 95% CI for the *β*_(PCAT total score)_ remained negative (Figure 3).

**Table 3:** General linear model analysis on treatment burden

	Unadjusted model		Adjusted model	
	*β* coefficient (95% CI)	*p*	*β* coefficient (95% CI)	*p*
Age, ≥65 years	−1.887 (−3.853 to 0.079)	.059	0.307 (−1.169 to 1.784)	.676
Gender, female	0.681 (−0.937 to 2.300)	.401	0.080 (−1.839 to 1.999)	.933
Education level, senior secondary school and above	−2.099 (−4.852 to 0.655)	.132	−0.388 (−2.692 to 1.915)	.735
Presence of social medical insurance	−1.571 (−4.303 to 1.161)	.253	1.205 (−1.070 to 3.480)	.290
Monthly income per head, ≥¥5000	0.414 (−3.538 to 4.367)	.834	−1.778 (−5.070 to 1.514)	.281
Duration of diseases, ≥5 years	2.171 (0.710–3.632)	.004	2.430 (0.808–4.051)	.004
Duration of CHC visits	−0.412 (−0.655 to −0.170)	.001	−0.084 (−0.339 to 0.170)	.506
Frequency of follow‐up	−2.186 (−2.948 to −1.424)	<.001	−1.046 (−1.609 to −0.483)	.001
Usual source of care, CHC	−6.364 (−9.587 to −3.141)	<.001	−3.681 (−6.284 to −1.077)	.007
Channels of consultations				
On‐site face‐to‐face visits to CHC	−5.389 (−7.905 to −2.873)	<.001	−0.208 (−3.585 to 3.170)	.902
Distance consultations	2.982 (0.748–5.216)	.010	1.711 (−1.434 to 4.855)	.278
Perceived positive role of the family doctor team	−3.651 (−6.275 to −1.027)	.007	−0.015 (−1.981 to 1.951)	.987
Number of healthcare needs	2.695 (2.139–3.251)	<.001	1.965 (1.384–2.545)	<.001
PCAT total score	−0.384 (−0.503 to −0.265)	<.001	−0.252 (−0.373 to −0.131)	<.001

Abbreviations: CHC, community health centre; CI, confidence interval; PCAT, Primary Care Assessment Tool.

## 4 DISCUSSION

### 4.1 Summary of the main findings

Our study demonstrated that in the absence of a secondary care gatekeeping function in primary care, approximately one third of multimorbid patients who had enrolled with the CHC family doctor team were still in favour of using specialist care regularly over primary care. They reported a significantly greater need for follow‐up care. Patients who had a family doctor team as the primary source of care reported better experiences with regard to first‐contact care, continuity of care, coordination of care, comprehensiveness of care and family centredness of care. Higher healthcare needs were significantly associated with increased treatment burden, whilst better patients' experiences were associated with lower treatment burden in the context of the family doctor team service delivery.

### 4.2 Strengths and limitations

We collected data from a relatively large sample of Chinese primary care service users with multimorbidity to understand the process of care using widely used international instruments with appropriate linguistic and psychometric validation. A focus on patients' experiences and process‐related treatment burden, rather than patients' satisfaction, could minimize subjective bias due to variations in patient‐level characteristics. A multistage sample design was accounted for to improve statistically valid inferences in this multisite study. However, several limitations should be mentioned. First, unmeasured confounders such as marital status, occupation, health‐related quality of life, so forth, could potentially influence the associations between patients' experiences and treatment burden, and causal inferences cannot be simply drawn using a cross‐sectional design. Second, data collected through patient self‐report were subject to recall bias. Inclusions of organization‐ or physician‐level questions pertaining to information on clinicians and personnel in the family doctor team could also be restricted. Third, another inherent limitation is that we were not able to use a more recently developed, multimorbidity‐oriented MTBQ^30^ to measure treatment burden owing to the timing of our project, despite evidence suggesting that the TBQ that we used in the present study was initially designed to measure treatment burden without restricting its scope to a single condition or treatment context.^20^, ^27^, ^28^, ^31^, ^32^, ^34^, ^35^ Fourth, we did not use outcome‐oriented biomedical indicators; instead, proxy measures representing key attributes of primary care were used from a process of care perspective. Last but not the least, the study participants were relatively wealthier than the general population, which may undermine the generalizability of findings. Given the healthcare utilization pattern in China,^9^ it is reasonable to assume that frequent users of primary care tend to be more prevalent in less affluent areas, where the strength of associations between patients' experiences and treatment burden might be stronger.

### 4.3 Comparison with the existing literature

Empirical evidence from low‐ and middle‐income countries suggests that one of the worst‐performing areas in primary care is the prevention and management of chronic diseases.^36^ Key problems commonly experienced by multimorbid patients included a lack of holistic care, poor service experiences and a high burden of disease treatment.^37^ This has also raised challenges in high‐income settings where previous work reported that unfavourable patients' experiences with primary care physicians were associated with a higher risk of hospitalization.^38^ This calls for a deserved attention to positive user experiences and competent care emphasizing healthcare needs and individual preferences, given the complexity of multimorbidity. The PCAT instrument measuring patients' experiences has been widely used^22^, ^39^, ^40^, ^41^, ^42^; however, most of these studies have assessed the process performance among service users overall, and research with a specific focus on attributes of primary care is lacking in the multimorbidity context. An understanding of process‐based measures as performance indicators is therefore of importance to inform areas for quality improvements in patient‐centred care.

As many clinical practice guidelines tend to focus on single conditions, the treatment burden was often assessed only as a subscale of specific disease scales or was considered only for the regimen associated with a particular condition.^20^, ^26^, ^27^ Despite varying approaches in the measurements, existing studies consistently reveal that higher levels of treatment burden relate to multimorbidity, access barriers, fragmented care and patient‐provider discordance.^43^, ^44^ Since the inception of the instrument in 2012, the TBQ has been widely used across 34 countries globally, including the United States, the United Kingdom, Canada, Australia, New Zealand, France, Switzerland, Spain and Qatar.^20^, ^27^, ^31^, ^32^, ^34^, ^35^ The established reliability and cross‐cultural adaptability of the TBQ to measure the burden of treatment in different populations and with various or multiple chronic conditions could justify the rationale of using the TBQ as a valid, reliable and internationally comparable instrument in our study. Since 2018, other similar tools have emerged for measuring multimorbidity‐related treatment burden, such as the MTBQ questionnaire, which was originally developed in elderly patients and may show the complexity of treatment burden from a more multimorbidity‐specific angle.^30^ However, it is worth noting that a comparison between different treatment burden measurements per se was not the aim of our study. Instead, we are more interested in determining whether primary care experiences were associated with treatment burden in our study population, which consisted of patients with two or more long‐term conditions. Given the proven ability of the TBQ to capture a comprehensive dimensionality of treatment burden, we believe that the relationship between patients' experiences and treatment burden observed in our study shall remain largely unchanged regardless of the instrument per se.

In our study, we found that longer duration of chronic diseases, greater healthcare needs and variables pertaining to the process of care, such as inadequate follow‐up and suboptimal primary care experiences, were associated with increased treatment burden. This could be explained by the speculation that patients' greater healthcare needs and extra efforts required to maintain their health may translate into additional workload, such as greater use of medications and challenges in behavioural modifications in coping with multimorbidity. Recent evidence suggests that across a wide range of health conditions and settings, a significant proportion of treatment burden results from the way in which healthcare is organized and delivered, rather than by specific patients, diseases or treatments.^20^ Our results confirmed that using primary care regularly and receiving frequently delivered follow‐up care with better patients' experiences correlated with alleviated treatment burden, which may imply a positive role of the primary care multidisciplinary team in the process of service delivery. This will also help contribute to the understanding of the extent to which routine interactions between patients and healthcare providers may impact on challenges that patients face in coping with multimorbidity in terms of processes of care.

Further, from a quantitative perspective, our sensitive analysis revealed that a higher primary care experience was consistently correlated with various components of lower treatment burden. This was particularly observed in alleviated burden with regard to doctor visits and relationships, schedule reorganization, administrative tasks, financial expenses, lifestyle changes and social and emotional impact. Similar aspects of treatment burden were identified in the previous literature as underlying factors of feeling ‘overburdened' with excessive healthcare workload, which included the following: (a) regular healthcare reminding patients of their health problems; (b) the financial burden of treatment; (c) the burden of arranging and adapting to medical appointments; and (d) difficulties in relationships with healthcare providers.^26^ These aspects of treatment burden are more closely related to the provision and uptake of routine healthcare services, with key clues to understanding the increases or decreases in levels of treatment burden that could be explained by the differences in the primary care context. It is, however, worth noting that not all treatment burden is avoidable,^45^ as unpleasant side effects may occur as a result of the prescriptions of multiple medications when indicated in complex clinical situations.

Patients in our study reported higher primary care assessment scores and lower treatment burden scores when directly compared to previous studies,^26^, ^27^, ^39^, ^40^ which may be due to the differences in study participants and settings where the availability, accessibility and acceptability of resources for primary care may differ. It might also be a reflection of potential gains from an improved process of care with the ‘family doctor team' that aims to translate key attributes of primary care into routine clinical practice.^16^ As an emerging service model of multidisciplinary team‐based care in China, the team provides continuous health maintenance as opposed to episodic treatment through the delivery of both patient‐centred and population‐oriented services following primary care principles. Healthcare professionals with a variety of expertise and skills can thus support a wider scope of community health services whilst alleviating the traditional workload of both GP clinicians and patients. The reshaped structure of service delivery with primary care as a trusted focal point may enable the personalization and prioritization of care to deliver what really matters to individual patients, taking into account their ability to manage complex conditions and circumstances. This helps strengthen and sustain relationships between patients and the care team, and is important for primary care to support the health of individuals with multimorbidity in the context of their life and community.^46^

Compared to the United Kingdom and other international countries where a primary care gatekeeping function is in place, the primary care facilities in China are still far from acting as a first‐contact point and regular source of care, despite ongoing development of the ‘family doctor team'. Concerns over the absence of healthcare gatekeeping and a rapid growth of hospital outpatients visits in China have been raised in the past decade.^2^, ^12^, ^47^, ^48^ The fact that patients can walk in directly to see a specialist doctor and receive medication prescriptions (i.e., without primary care referral) has led to overutilization of outpatient specialist care services at secondary/tertiary hospitals in China for many years. A previous review based on national data suggested a widening gap between hospital outpatient care and primary care settings in total person‐time of diagnosis and treatment.^12^ A recent World Bank/WHO report also showed that the share of outpatient services in hospitals has increased from 34.9% to 39.1%, while the proportion in primary care facilities has decreased from 61.9% to 57.4% since 2010.^48^ In our study, up to one third of multimorbid patients enrolled with the CHC family doctor team were still in favour of using specialist care regularly over primary care. The proportion was indeed lower than previously reported in a large population‐based cross‐sectional study conducted by us earlier in the same study region, where around 43.7% of multimorbid patients considered the hospital outpatient services as the usual source of healthcare or had mixed utilization preferences.^2^ We speculate that the implementation of a primary care team may play a role in improving patients' experiences with primary care, associated with alleviated treatment burden that has been shown in this study, and therefore may enhance people's engagement with and confidence in routine primary care.

### 4.4 Implications for research and practice

Primary care is expected to lie at the heart of care delivery in response to healthcare needs from patients with multimorbidity. The family doctor team initiative shares similarities to the GP service delivery in the United Kingdom with regard to strengthened relationships between the enrolled individual patients and the primary care providers in a continuous and collaborative manner. This, of course, requires proper knowledge, adequate skills, right professional values and positive attitudes that embrace core attributes of primary care. Our study adds to the evidence favouring the benefits of primary care multidisciplinary team‐based service delivery in coping with multimorbidity. This carries international implications for other countries where primary care transformation is in progress as a vital step towards achieving an improved team‐based approach to population health and multimorbidity management. Further longitudinal investigations are warranted to examine the effects of the family doctor team service delivery on the care for patients with multimorbidity in the long term. Efforts to explore an optimal panel size of registration per primary care team and the ratio of GP clinicians versus allied healthcare professionals linked with health outcomes may also help to inform strategies for multimorbidity care over time in areas of different socioeconomic strata.

It has been widely recognized that treatment burden prevents optimal adherence to the provision and management of care for people with long‐term conditions,^21^, ^35^ and thus may reduce the overall effectiveness of the health system. A deeper understanding of the complexities of care experiences and the manifestations of treatment burden will help inform an integrated approach at both practice and policy levels to improved chronic care and service delivery in primary care.^49^ Our data suggested that an optimal profile of patients' experiences and lower treatment burden were more likely to be seen in multimorbid patients who used the family doctor team as the usual source of primary care. This may lay the foundation for future work to explore the long‐term benefits of improved adherence to clinical recommendations and the potential impact of active participation in multimorbidity care plans on desired health outcomes.

## 5 CONCLUSION

Our study suggested that higher healthcare needs were significantly associated with increased treatment burden, whilst better patients' experiences were associated with lower treatment burden in the context of the family doctor team service delivery. This implies the necessity of optimizing the key attributes of primary care in person‐centred service delivery and quality improvement, and is therefore of major relevance to healthcare strategies aiming to deliver less burdensome care for people with multimorbidity.

## CONFLICT OF INTERESTS

The authors declare that there are no conflict of interests.

## AUTHOR CONTRIBUTIONS

Harry H. X. Wang and Xiu‐Jing Hu conceived the idea of the study. Harry H. X. Wang, Yu‐Ting Li and Jia‐Ji Wang participated in the fieldwork coordination. Data analysis was conducted by Xiu‐Jing Hu and Yu‐Ting Li. All authors contributed to the literature search and interpretation of the data. Harry H. X. Wang and Xiu‐Jing Hu wrote the first draft. All authors have read, contributed to and approved the final version of the manuscript.

## Data Availability

The data that support the findings of this study are available from the corresponding author upon reasonable request.
